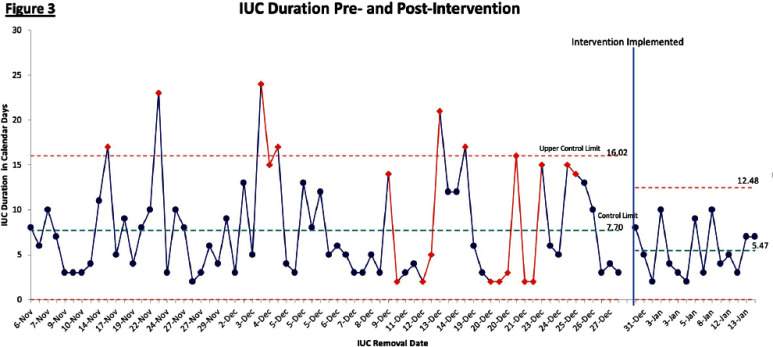# Utilizing a Process Improvement Approach and Implementing a Plan-Do-Study-Act (PDSA) Cycle to Decrease CAUTIs on a Cardiology Unit

**DOI:** 10.1017/ash.2025.395

**Published:** 2025-09-24

**Authors:** Akanksha Arya, Owen Renault, William Eissler, Kathryn DiMartino

**Affiliations:** 1 Massachusetts General Hospital/ Brigham and Women’s Hospital; 2 Newton-Wellesley Hospital; 3 Mass General Brigham Newton-Wellesley Hospital; 4 Newton Wellesley Hospital

## Abstract

**Background:** There is a high prevalence of catheter associated urinary tract infections (CAUTIs) on a hospital cardiology unit, with a rate of 2.48 CAUTIs per 1,000 catheter days over the past two years compared to the national average of 0.96 CAUTIs for similar units. CAUTIs lead to increased lengths of stay, mortality, and hospital expenditures. Per NHSN, the presence of an indwelling urinary catheter (IUC) increases the risk for developing a CAUTI by 3-7% each day an IUC is in place. **Method:** A process improvement approach was utilized to study the problem of increased CAUTIs and implement a PDSA intervention.

A process map was created to identify opportunities for error that could increase risk for CAUTIs (Figure 1). Contributing factors were explored through developing a driver diagram (Figure 2).

Data was collected to study root causes of CAUTI development and identify opportunities for improvement. 7 nurses were observed placing IUCs in mannequins to assess insertion practices. 19 maintenance audits of IUCs among patients were conducted. Electronic medical record (EMR) data was compiled to assess hospital location of catheter insertion, catheter utilization ratio, indication for insertion, and duration of catheterization. Based on data, team decided to focus PDSA intervention on reducing IUC duration, a process measure for the desired outcome of reducing CAUTIs. **Results:** EMR baseline data during the period 11/6/2024- 12/29/2024 revealed an average IUC duration of 7.92 days. A SMART(IE) goal was established to reduce the average duration of IUCs on this unit by 15% from 7.92 days to 6.73 days within 4 weeks.

An intervention was developed to incorporate discussion of IUC indication, duration, and eligibility for removal for patients with IUCs during daily multidisciplinary rounds. Unit charge nurses received training on CAUTI prevention, facilitating rounds discussions, and data collection. Intervention is being implemented over the period 12/30/2024- 1/25/2025.

During the pre-intervention period 11/6/2024- 12/29/2024, 70 IUCs were reviewed. In preliminary analysis of the post-intervention period of 12/30/24- 1/15/25, 15 IUCs were reviewed. Preliminary analysis shows the average duration of IUCs per patient decreased by 31%, to an average of 5.47 days (Figure 3). There were 4 IUCs that were removed after discussions at multidisciplinary rounds. **Conclusion:** Process improvement tools can be utilized to study contributors to CAUTIs and develop unit-level solutions. Preliminary data demonstrates that incorporating review of IUCs during multidisciplinary rounds may reduce average duration of IUC use.

**Figure:**
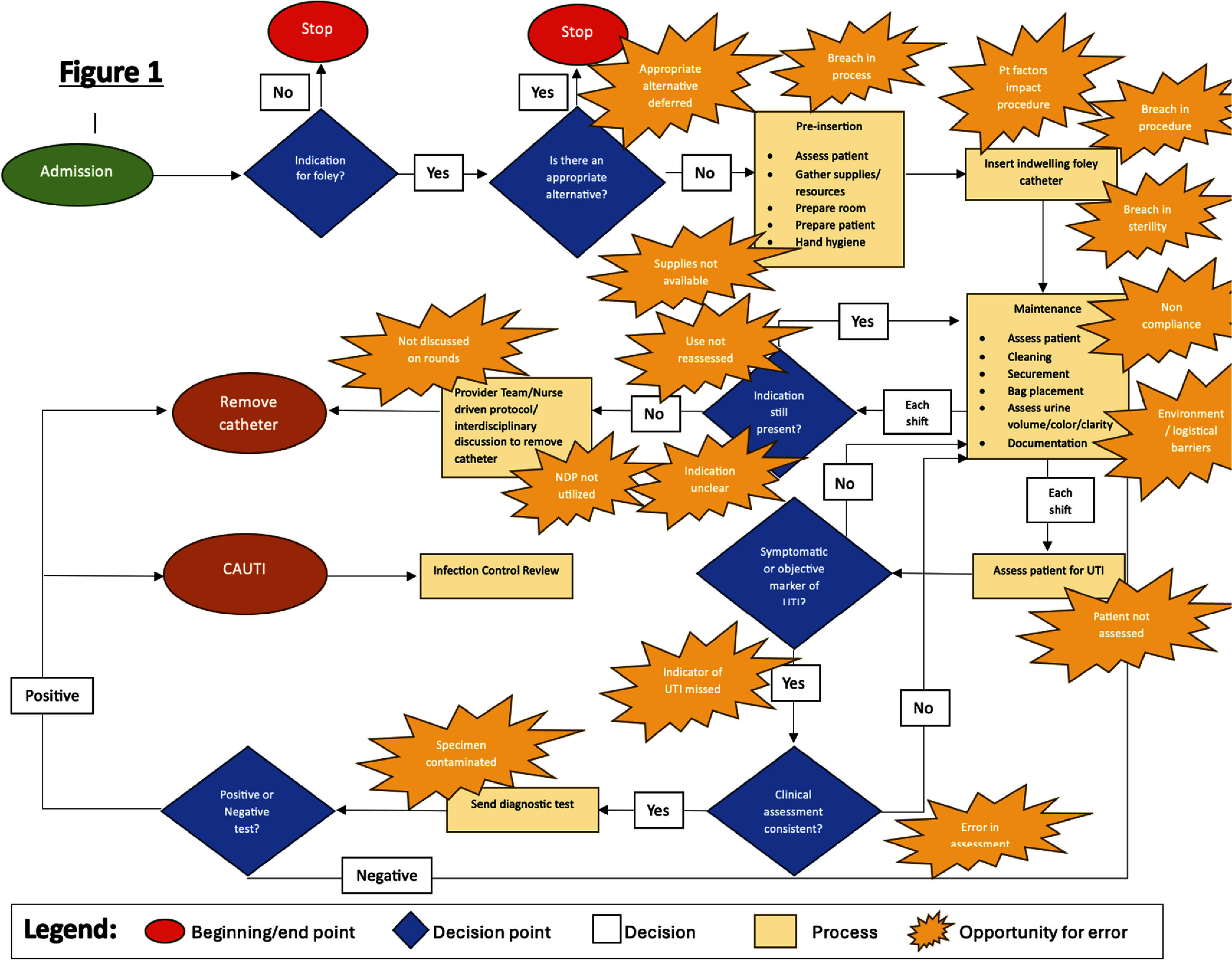

**Figure:**
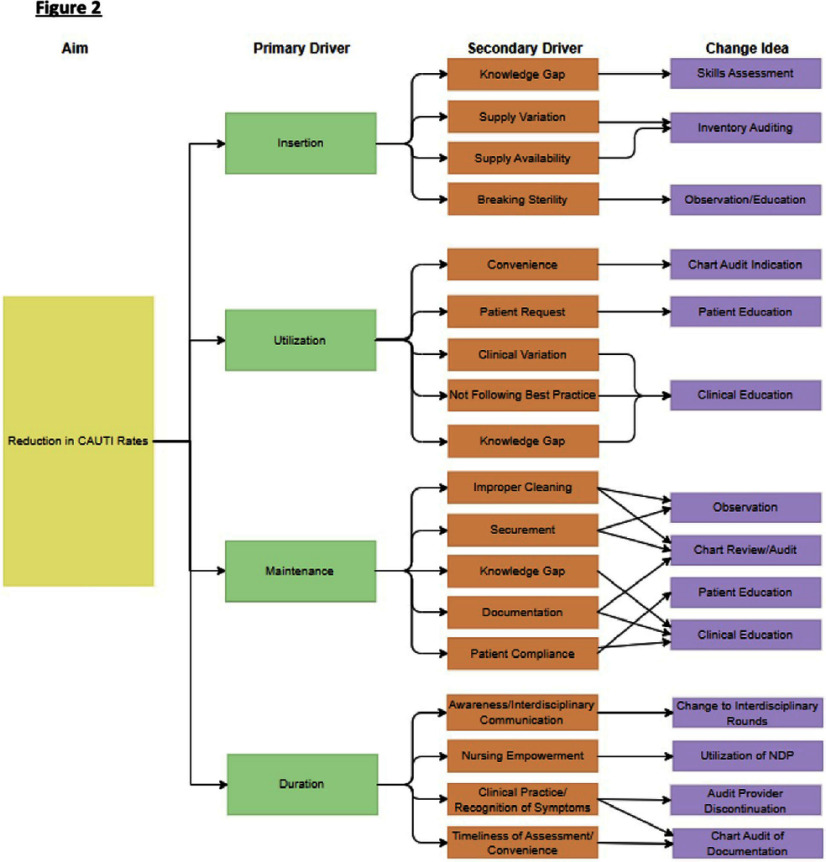

**Figure:**